# Newly diagnosed diabetes has high risk for cardiovascular outcome in ischemic stroke patients

**DOI:** 10.1038/s41598-021-92349-y

**Published:** 2021-06-21

**Authors:** Kyung-Hee Cho, Sun U. Kwon, Ji Sung Lee, Sungwook Yu, A-Hyun Cho

**Affiliations:** 1 Department of Neurology, Korea University Anam Hospital, Korea University College of Medicine, Seoul, South Korea; 2 Department of Neurology, Asan Institute for Life Sciences, Asan Medical Center, University of Ulsan College of Medicine, Seoul, South Korea; 3 Clinical Research Center, Asan Institute for Life Sciences, Asan Medical Center, University of Ulsan College of Medicine, Seoul, South Korea; 4 Department of Neurology, Yeouido St. Mary’s Hospital, College of Medicine, The Catholic University of Korea, Seoul, South Korea

**Keywords:** Neuroscience, Neurology

## Abstract

We investigated cardiovascular outcomes in ischemic stroke patients with newly diagnosed diabetes mellitus (DM) compared with those of patients with previously known DM and no DM using the glycosylated hemoglobin (HbA1c) criteria. The relationship between new DM diagnosis and cardiovascular risk remains unclear to date. We performed post hoc analysis using the data of participants from the Prevention of Cardiovascular events in iSchemic Stroke patients with high risk of cerebral hemOrrhage (PICASSO) trial. Newly diagnosed DM was defined as HbA1c of ≥ 6.5% without known DM history. The outcome was the incidence of composite cardiovascular events, including stroke (ischemic and hemorrhagic), myocardial infarction, and cardiovascular death. In total, 1306 patients were included; 38 patients (2.9%) had newly diagnosed DM; 438 patients (33.5%), known DM; and 830 patients (63.6%), no DM. In patients with newly diagnosed DM, known DM, and no DM, the incidence of ischemic stroke was 8.93, 3.79, and 2.64 per 100 person-years (log-rank test; p = 0.0092), while that of composite cardiovascular events was 8.93, 5.92, and 3.87 per 100 person-years (p = 0.025), respectively. Newly diagnosed DM was an important risk factor for ischemic stroke and composite cardiovascular events after ischemic stroke.

**Registration:** URL: https://www.clinicaltrials.gov. Unique identifier: NCT01013532.

## Introduction

The incidence of cardiovascular disease is approximately two-fold higher in people with diabetes mellitus (DM), even after adjusting for other established cardiovascular risk factors^1^. Cardiovascular risk reduction is a treatment goal in DM patient management. Furthermore, DM is an important risk factor for cardiovascular outcomes after ischemic stroke^2–4^.

There is limited data on cardiovascular outcomes of newly diagnosed DM in ischemic stroke patients without DM history. A hospital-based prospective cohort study conducted in 701 Cameroonian patients with acute stroke showed that newly diagnosed DM patients had a more severe stroke at the time of admission and a higher incidence of mortality at 3 months after stroke than the patients with known DM^5^. However, this study included both ischemic and hemorrhagic stroke patients and had a short follow-up period. Additionally, the endpoints of this study were modified Rankin Scale (mRS) scores and 3-month mortality. Among 1251 patients with acute ischemic stroke in a previous Chinese study, newly diagnosed DM was an independent risk factor for 1-year mortality, stroke recurrence, and poor functional outcome (mRS ≥ 3)^6^. However, 1-year prognosis of newly diagnosed DM patients was assessed through telephone follow-ups, and all outcome variables were compared with patients with no DM history rather than those with known DM history.

We aimed to evaluate the relationship between newly diagnosed DM and long-term prognosis of patients with acute ischemic stroke and determine whether newly diagnosed DM was associated with a higher risk of cardiovascular outcomes after ischemic stroke as opposed to known or no DM history.

## Results

Among the 1534 patients randomized in the PICASSO trial, 1306 (85.1%) patients whose initial HbA1c level had been recorded were included in the present study. The mean age of the patients was 66.2 ± 10.7 years and 503 (38.5%) were females. Of the 1306 patients, the qualifying ischemic events were ischemic stroke in 1248 patients (95.6%) and transient ischemic attack in 58 patients (4.4%). Patients were followed-up for a median duration of 2.0 years (IQR 1.0–3.0).

Of these 1306 patients, 38 (2.9%) were classified as patients with newly diagnosed DM, 438 (33.5%) as patients with known DM history, and 830 (63.6%) as patients with no DM history. The demographic and clinical characteristics of these three groups are summarized in Table 1. History of hypertension (p = 0.0266), history of dyslipidemia (p = 0.0003), body weight (p = 0.0292), waist circumference (p = 0.0017), body mass index (p = 0.0022), initial glucose level (p < 0.0001), triglyceride level (p = 0.0011), HDL level (p < 0.0001), HbA1c level (p < 0.0001), initial NIHSS scores (p = 0.0019), and initial mRS scores (p = 0.0072) were significantly different among the three groups. Newly diagnosed DM patients had a higher body weight, waist circumference, BMI, and initial triglyceride level than those with known or no DM history. Patient with known DM were more likely to have hypertension or dyslipidemia as well as higher NIHSS scores, glucose level, and HbA1c levels. However, systolic and diastolic blood pressure, history of coronary artery disease, current smoking status, low-density lipoprotein cholesterol (LDL) level, and education level did not differ among the three groups.

**Table 1:** Demographic and clinical characteristics according to diabetes status.

	No DM (n = 830)	Known DM (n = 438)	Newly diagnosed DM (n = 38)	*p* value
Age (years)	66.1 ± 11.1	66.4 ± 9.9	64.7 ± 10.9	0.6007
Sex, male	521 (62.8)	256 (58.4)	26 (68.4)	0.2167
Education (years)	9 (6–12)	9 (6–12)	9 (6–12)	0.5729
**Qualifying ischemic event**				0.4464
Ischemic stroke	789 (95.1)	423 (96.6)	36 (94.7)	
Transient ischemic attack	41 (4.9)	15 (3.4)	2 (5.3)	
**Medical history**				
Hypertension	722 (87.0)	403 (92.0)	34 (89.5)	0.0266
Dyslipidemia	310 (37.3)	215 (49.1)	15 (39.5)	0.0003
Current smoking	38 (4.6)	24 (5.5)	4 (10.5)	0.2313
Coronary artery disease	176 (21.2)	79 (18.0)	12 (31.6)	0.0929
Family history of stroke	167 (20.1)	83 (18.9)	9 (23.7)	0.7362
**Vital signs**				
Systolic blood pressure (mmHg)	134.8 ± 18.6	136.1 ± 18.4	134.8 ± 15.6	0.5042
Diastolic blood pressure (mmHg)	80.3 ± 11.9	80.0 ± 11.7	77.2 ± 10.7	0.2677
Height (cm)	161.4 ± 9.3	161.3 ± 8.6	161.8 ± 8.3	0.9377
Body weight (kg)	63.1 ± 11.9	64.4 ± 11.5	67.4 ± 13.9	0.0292
Waist circumference (cm)	87.2 ± 9.5	89.1 ± 9.6	89.9 ± 9.3	0.0017
Body mass index (kg/m^2^)	24.1 ± 3.4	24.7 ± 3.5	25.7 ± 4.6	0.0022
**Laboratory findings (mg/dL)**				
Glucose	105.0 ± 23.5	150.5 ± 60.7	125.4 ± 26.7	< 0.0001
Total cholesterol	168.6 ± 40.4	166.2 ± 43.1	168.2 ± 36.7	0.6334
Triglyceride	122.8 ± 86.8	140.0 ± 84.9	152.1 ± 106.6	0.0011
HDL	46.2 ± 11.9	43.0 ± 12.3	47.7 ± 10.6	< 0.0001
LDL	102.2 ± 34.1	102.8 ± 39.5	98.5 ± 29.4	0.7771
HbA1c (%)	5.65 ± 0.38	7.24 ± 1.42	6.78 ± 0.37	< 0.0001
hsCRP	0.24 ± 0.31	0.28 ± 0.38	0.28 ± 0.34	0.1019
Baseline NIHSS score	1 (0–3)	2 (1–3)	1 (0–3)	0.0190
**Baseline mRS score**				0.0072
0	151 (18.3)	53 (12.2)	10 (26.3)	
1	345 (41.8)	171 (39.4)	16 (42.1)	
2	187 (22.7)	101 (23.3)	6 (15.8)	
3	81 (9.8)	51 (11.8)	2 (5.3)	
4	54 (6.5)	51 (11.8)	4 (10.5)	
5	7 (0.8)	7 (1.6)	0 (0.0)	

Values are presented as mean ± standard deviation, number (%), or median (interquartile range).

*DM* diabetes mellitus, *HDL* high-density lipoprotein cholesterol, *LDL* low-density lipoprotein cholesterol, *hsCRP* high-sensitivity C-reactive protein, *NIHSS* National Institute of Health Stroke Scale, *mRS* modified Rankin Scale.

Table 2 shows the summary of all events occurring during 2 years of follow-up. Of 830 patients without DM, 438 patients with known DM, and 38 patients with newly diagnosed DM, composite cardiovascular events occurred in 66 (7.9%), 50 (11.4%), and 6 (15.8%) patients, respectively; the incidence per 100 person-years in patients with no DM history, with known DM history, and newly diagnosed DM was 3.87, 5.92, and 8.93, respectively (p = 0.025). The incidence of any stroke (p = 0.032) and ischemic stroke (p = 0.009) events progressively increased from patients with no DM to those with newly diagnosed DM, and this difference was statistically significant. Both cerebral hemorrhage event and ischemic stroke event occurred in a patient with known DM.

**Table 2:** Comparison of incidence rate among diabetes status.

	No DM	Known DM	Newly diagnosed DM	*p* value^†^
**Composite cardiovascular events**				0.0252
No. of event	66	50	6	
Incidence rate per 100 person-years (95% CI)	3.87 (3.04–49.20)	5.92 (4.49–78.16)	8.93 (4.01–198.74)	
**Cerebral hemorrhage**				0.4332
No. of event	13	10	0	
Incidence rate per 100 person-years (95% CI)	0.76 (0.44–13.10)	1.18 (0.64–21.98)	0.00 (0.00–0.00)	
**Stroke**				0.0320
No. of event	58	41	6	
Incidence rate per 100 person-years (95% CI)	3.40 (2.63–43.94)	4.86 (3.58–65.96)	8.93 (4.01–198.74)	
**Ischemic stroke**				0.0092
No. of event	45	32	6	
Incidence rate per 100 person-years (95% CI)	2.64 (1.97–35.29)	3.79 (2.68–53.60)	8.93 (4.01–198.74)	
**Myocardial infarction**				0.0852
No. of event	3	6	0	
Incidence rate per 100 person-years (95% CI)	0.18 (0.06–5.44)	0.71 (0.32–15.80)	0.00 (0.00–0.00)	
**Cardiovascular death**				0.4519
No. of event	5	5	0	
Incidence rate per 100 person-years (95% CI)	0.29 (0.12–7.03)	0.59 (0.25–14.21)	0.00 (0.00–0.00)	

*DM* diabetes mellitus, *CI* confidence interval.

^†^p-value by log rank test.

Table 3 shows the HR for cardiovascular outcomes according to the patients’ diabetes status. In comparison with the patients with no DM history, the adjusted HR for composite cardiovascular outcomes in patients with newly diagnosed DM was 2.36 (95% CI 1.01–5.54; p = 0.0480). On comparing stroke events between patients with no DM history and those with newly diagnosed DM, the adjusted HR of the latter group of patients was found to be 2.72 (95% CI 1.15–6.43; p = 0.0223). The adjusted HR for ischemic stroke events in patients with newly diagnosed DM was 3.34 (95% CI 1.39–8.01; p = 0.0069) when compared with those with no DM history. Only ischemic stroke events were found to be significantly different between the patients with newly diagnosed DM and known DM history (adjusted HR 2.47; 95% CI 1.00–6.10; p = 0.0499). Newly diagnosed DM was independently associated with the occurrence of composite cardiovascular events, stroke, and ischemic stroke.

**Table 3:** Hazard ratios and 95% Confidence Intervals for cardiovascular outcome by diabetes status.

	Unadjusted HR (95% CI)	*P* value	Adjusted HR (95% CI)	*p* value
**Composite cardiovascular events**				
**DM status**		0.0279		0.0480
No DM (reference)				
Known DM	1.50 (1.04–2.16)	0.0317	1.49 (0.98–2.28)	0.0639
Newly diagnosed DM	2.32 (1.01–5.35)	0.0485	2.36 (1.01–5.54)	0.0480
**Cerebral hemorrhage**				
**DM status**		0.5765		0.7492
No DM (reference)				
Known DM	1.55 (0.68–3.57)	0.2991	1.45 (0.55–3.79)	0.4501
Newly diagnosed DM	0.97 (0.05–17.84)	0.9824	1.03 (0.05–20.04)	0.9827
**Stroke**				
**DM status**		0.0374		0.0506
No DM (reference)				
Known DM	1.39 (0.93–2.07)	0.1061	1.37 (0.87–2.18)	0.1784
Newly diagnosed DM	2.65 (1.14–6.13)	0.0233	2.72 (1.15–6.43)	0.0223
**Ischemic stroke**				
**DM status**		0.0136		0.0233
No DM (reference)				
Known DM	1.39 (0.89–2.19)	0.1519	1.35 (0.80–2.29)	0.2608
Newly diagnosed DM	3.39 (1.45–7.96)	0.0049	3.34 (1.39–8.01)	0.0069
**Myocardial infarction**				
**DM status**		0.1769		0.1505
No DM (reference)				
Known DM	3.75 (0.92–15.27)	0.0652	4.62 (0.99–21.66)	0.0521
Newly diagnosed DM	3.51 (0.14–86.51)	0.4425	2.08 (0.07–65.73)	0.6777
**Cardiovascular death**				
**DM status**		0.5242		0.8301
No DM (reference)				
Known DM	2.02 (0.57–7.19)	0.2756	1.41 (0.31–6.30)	0.6569
Newly diagnosed DM	2.34 (0.10–52.24)	0.5914	2.27 (0.09–57.70)	0.6202

Hazard ratio adjusted for hypertension, dyslipidemia, body mass index, Glucose, Triglyceride, HDL and NIHSS score.

*DM* Diabetes mellitus, *HR* hazard ratio, *CI* confidence interval.

**Figure 1:**
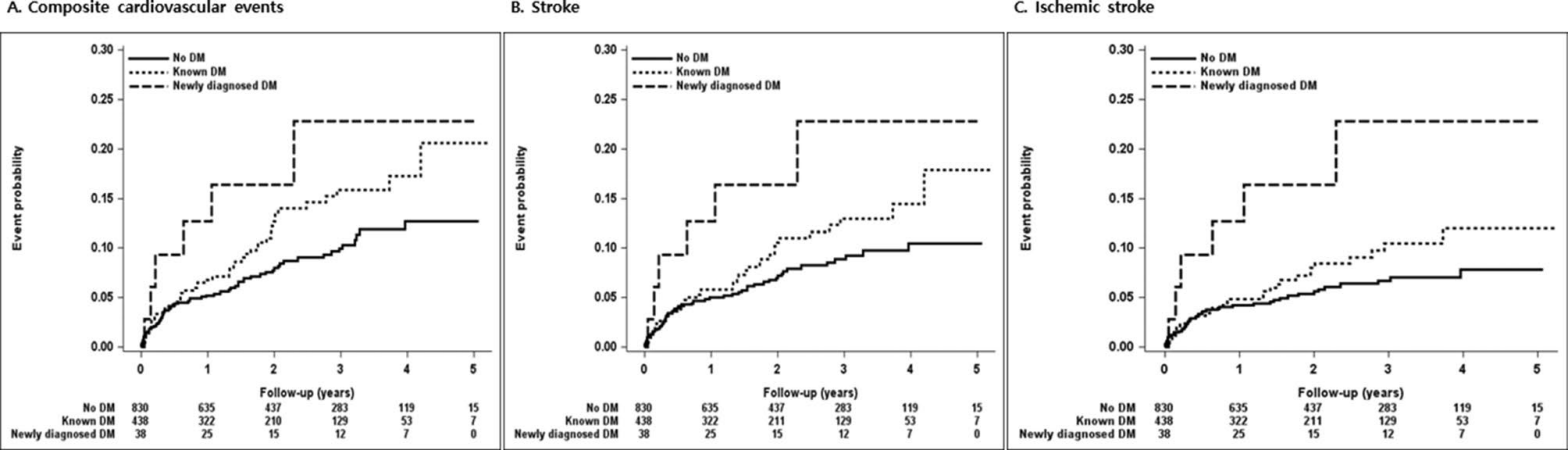
Kaplan–Meier estimates of event probability expressed as diabetes status.

Figure 1 depicts Kaplan–Meier curves for composite cardiovascular events (Fig. 1A), stroke (Fig. 1B), and ischemic stroke (Fig. 1C) among the three groups. Outcome event probability was significantly different among the three groups and progressively increased from patients with no DM history to those with newly diagnosed DM. Notably, patients with newly diagnosed DM had a higher risk of cardiovascular outcomes than the patients with known DM history and those with no DM history.

## Discussion

The present study found that the incidence of composite cardiovascular events after ischemic stroke significantly is higher in patients with newly diagnosed DM. Especially, the incidence of ischemic stroke is higher in patients with newly diagnosed DM compared with the incidence in both the patients with known and no DM history, after adjusting for hypertension, dyslipidemia, BMI, glucose level, triglyceride level, HDL level, and NIHSS score. Patients with known DM had a higher incidence of hypertension and dyslipidemia as well as higher initial NIHSS scores and mRS scores compared with the patients with newly diagnosed DM and no DM history. Moreover, the initial glucose and HbA1c levels were significantly higher in the patients with known DM history. Contrastingly, newly diagnosed DM patients had a significantly higher body weight, longer waist circumference, higher BMI, and higher triglyceride level. Presence of newly diagnosed, undetected DM in patients with ischemic stroke supports the hypothesis that unrecognized metabolic abnormalities contribute to adverse cardiovascular outcomes. Metabolic syndrome symptoms, including obesity and high serum triglyceride level, appears early during the course of the disease and several years prior to diabetes development. Obesity parameters (such as BMI and waist circumference) and higher triglyceride level of the patients with newly diagnosed DM in our study were consistent with previous studies which demonstrated significant associations between obesity parameters and risk for diabetes^7,8^. Therefore, long-term glucometabolic derangement in subdiabetic range may become a risk factor for cardiovascular disease and therefore influence its outcomes^9^.

It is well recognized that an elevated HbA1c level and hyperglycemia are risk factors for the development of cardiovascular events^10^. Previous studies have shown an increased incidence of adverse events in patients with non-diabetic myocardial infarction who had an elevated HbA1c level^9^, suggesting that the underlying mechanisms do not entirely pertain to stress-induced hyperglycemia. In the present study, we also observed that known DM patients with higher HbA1c and glucose levels had more severe symptoms initially. However, newly diagnosed DM is associated with increased incidence of cardiovascular events including ischemic stroke despite adequate secondary prevention with antiplatelet agents. Routine HbA1c measurement in acute stroke patients may facilitate the identification of patients with undetected DM or those who are at an increased risk of DM development in the future^11^.

To our knowledge, this is the first and the largest study that uses HbA1c level to assess the effect of newly diagnosed DM on prognosis after ischemic stroke and compares the effect with that associated with both known and no DM history. Although previous studies suggested that newly diagnosed DM was associated with poor prognosis after stroke^5,6^, neither study separately reported the adjusted HRs for cardiovascular events in patients with known DM and newly diagnosed DM. Thus, from previous studies, it was unclear whether newly diagnosed DM affected prognosis; however, our study clearly demonstrated that newly diagnosed DM independently and adversely affects prognosis after ischemic stroke. The main strength of this study is a homogeneous patient group because only non-cardioembolic patients who had imaging features of small vessel occlusive disease were included.

A very small proportion (2.9%, 38 of 1306) of patients from our PICASSO database who did not have pre-existing DM but were admitted owing to ischemic stroke were newly diagnosed with DM as opposed to previous studies, which reported a higher prevalence of newly diagnosed DM in the range of 9.4–43.1% among stroke patients^5,6,12,13^. This discrepancy could be caused by the differences in study design, the study area, the education level of patients, and public awareness of diabetes. South Korea has a well-organized health care system, and 97.2% of the Korean population are covered by the National Health Insurance Service, which provides regular health check-ups every 1 or 2 years^14^.

Our study has several limitations. First, it was a retrospective sub-analysis of a prospective study. Moreover, it has the potential to be affected by chance and confounding bias that exist in a post hoc design, even if adjustment analyses are performed. Second, there were only a small number of patients with newly diagnosed DM, which reduced the statistical power to draw definite conclusions. Third, although the main trial was performed across three Asian countries, this analysis included only Korean patients owing to unavailable data. Thus, caution should be exercised while generalizing this result to all patients with stroke. Finally, although newly diagnosed DM was defined as initial HbA1c level of ≥ 6.5% without a history of DM in this study, in the original study, newly diagnosed DM patients had been categorized into the no DM history group. We had no data regarding new occurrences of DM or the initiation of glucose-lowering medication during the follow-up period.

## Conclusions

Our study found that the occurrence of composite cardiovascular events after ischemic stroke increases in newly diagnosed DM patients. Furthermore, newly diagnosed DM may pose higher risk of recurrent cerebral infarction in ischemic stroke patients than that the risk posed by known DM history. Further research is needed to elucidate how newly diagnosed DM might affect cardiovascular outcome. Routine HbA1c measurement in ischemic stroke may help identify patients with undetected DM or prediabetic patients having an increased risk of DM development in the future.

## Methods

### Study design and patients

We performed post hoc analysis by analyzing the data of participants from the Prevention of CArdiovascular events in iSchemic Stroke patients with a high risk of cerebral hemOrrhage (PICASSO) trial. Patients were enrolled into the PICASSO study within 6 months of experiencing ischemic stroke or transient ischemic attack and had symptomatic or asymptomatic ICH or multiple cerebral microbleeds^15^. It was a two-by-two factorial study designed to determine the efficacy and safety of cilostazol and probucol. Between April 2010 and August 2015, and was performed to investigate which antiplatelet agent may be more effective and safe to treat ischemic stroke patients with high risk of cerebral hemorrhage. Conclusionally, cilostazol was non-inferior to aspirin for the prevention of cardiovascular events, but did not reduce the risk of hemorrhagic stroke. Details of the rationale, study design, characteristics of the participants, and principal results of PICASSO trial have been published elsewhere^15,16^. All methods were carried out in accordance with relevant guidelines (STROBE guidelines). All participants provided written informed consent and the trial was approved by the institutional review board (IRB) of all participating centers. (Asan medical center IRB No. 2009–0189). Among them, patients whose initial glycosylated hemoglobin (HbA1c) level had been recorded were included in the present analysis. There were no other exclusion criteria.

Clinical information was collected at baseline. The data included age, sex, education, known medical history (the presence of hypertension, diabetes, dyslipidemia, coronary artery disease, and current smoking status), family history of stroke, use of DM medication, initial National Institutes of Health stroke scale (NIHSS) score, initial mRS score, vital signs, body mass index (BMI), and blood test results (including glucose, HbA1c, lipid profile). HbA1c level was obtained in screening period before randomization in PICASSO trial.

### Subgroups and definitions

For this analysis, PICASSO trial participants whose initial HbA1c level was recorded were classified either as no DM, known DM, or newly diagnosed DM. Known DM was established on the basis of history if the patient had been informed of the diagnosis by a physician before the index stroke or was taking oral hypoglycemic agents or insulin or receiving dietary therapy. Newly diagnosed DM was defined as an initial HbA1c cutoff value of ≥ 6.5% as per the recommendation by the American Diabetes Association, without a history of DM because the current guidelines support the use of HbA1c for the diagnosis of diabetes.

### Outcome measures

The primary and secondary outcomes were prespecified at the start of the PICASSO trial^15^. All outcomes were confirmed by the Central Independent Adjudication Committee. The outcome of the present study was the occurrence of composite cardiovascular events, including stroke (ischemic and hemorrhagic), myocardial infarction, and cardiovascular death.

### Statistical analysis

Differences in baseline characteristics among the three groups were compared. The frequency (percentage), mean (± standard deviation, SD), or median (interquartile range, IQR) are reported depending on the variable type. Categorical variables were analyzed using Pearson’s chi-square test or Fisher’s exact test, and continuous variables were analyzed using Analysis of Variance (ANOVA) and Kruskal–Wallis tests, as appropriate. The incidence rates of outcomes were calculated by dividing the number of incident cases with the total follow-up period (person-years). Cumulative events of outcomes were assessed using Kaplan–Meier estimates and compared using the log-rank test. Hazard ratios (HRs) and 95% confidence interval (CI) values of outcomes were analyzed using the Cox proportional hazards model for the three groups after adjusting for hypertension, dyslipidemia, BMI, glucose level, triglyceride level, high-density lipoprotein cholesterol (HDL) level, and NIHSS score. We tested the assumption of proportionality of hazards using the numerical method proposed by Lin et al. derived from the cumulative sum of Martingale residuals^17^. We found no evidence for the violation of the proportional hazards assumption. All reported p-values were two-sided and not adjusted for multiple testing. All statistical results were analyzed using SAS 9.4 (SAS Institute Inc., Cary, NC, USA), and p-values of < 0·05 were considered to be statistically significant.

### Ethics approval and consent to participate

The study protocol was approved by the Institutional review board or ethics committee at each participating hospital. All patients provided written informed consent for participation in the trial.

### Consent for publication

The authors have reviewed the manuscript and consent for publication.

## Data Availability

The data of this study may be available on reasonable request to the PICASSO Trial Registry.

## Abbreviations

DM: Diabetes mellitus
PICASSO: Prevention of CArdiovascular events in iSchemic Stroke patients with a high risk of cerebral hemorrhage
HbA1c: Glycosylated hemoglobin
NIHSS: National Institutes of Health stroke scale
BMI: Body mass index
HR: Hazard ratio
CI: Confidence interval
HDL: High-density lipoprotein cholesterol
LDL: Low-density lipoprotein cholesterol
